# Rice with reduced stomatal density conserves water and has improved drought tolerance under future climate conditions

**DOI:** 10.1111/nph.15344

**Published:** 2018-07-24

**Authors:** Robert S. Caine, Xiaojia Yin, Jennifer Sloan, Emily L. Harrison, Umar Mohammed, Timothy Fulton, Akshaya K. Biswal, Jacqueline Dionora, Caspar C. Chater, Robert A. Coe, Anindya Bandyopadhyay, Erik H. Murchie, Ranjan Swarup, W. Paul Quick, Julie E. Gray

**Affiliations:** ^1^ Department of Molecular Biology and Biotechnology University of Sheffield Sheffield S10 2TN UK; ^2^ International Rice Research Institute DAPO 7777 Metro Manila Philippines; ^3^ Division of Plant and Crop Science University of Nottingham, Sutton Bonington Campus Loughborough LE12 5RD UK; ^4^ Department of Genetics University of Cambridge Cambridge CB2 3EH UK; ^5^ Department of Biology University of North Carolina at Chapel Hill Chapel Hill NC 27599‐3280 USA; ^6^ Departamento de Biología Molecular de Plantas Instituto de Biotecnología Universidad Nacional Autónoma de Mexico Cuernavaca 62210 Mexico; ^7^ ARC Centre of Excellence for Translational Photosynthesis Australian National University Canberra ACT 2601 Australia

**Keywords:** climate change, drought, epidermal pattering factor, heat stress, rice, stomata, water conservation

## Abstract

- Much of humanity relies on rice (*Oryza sativa*) as a food source, but cultivation is water intensive and the crop is vulnerable to drought and high temperatures. Under climate change, periods of reduced water availability and high temperature are expected to become more frequent, leading to detrimental effects on rice yields.
- We engineered the high‐yielding rice cultivar ‘IR64’ to produce fewer stomata by manipulating the level of a developmental signal. We overexpressed the rice epidermal patterning factor *OsEPF1*, creating plants with substantially reduced stomatal density and correspondingly low stomatal conductance.
- Low stomatal density rice lines were more able to conserve water, using *c*. 60% of the normal amount between weeks 4 and 5 post germination. When grown at elevated atmospheric CO_2_, rice plants with low stomatal density were able to maintain their stomatal conductance and survive drought and high temperature (40°C) for longer than control plants. Low stomatal density rice gave equivalent or even improved yields, despite a reduced rate of photosynthesis in some conditions.
- Rice plants with fewer stomata are drought tolerant and more conservative in their water use, and they should perform better in the future when climate change is expected to threaten food security.

## Introduction

The combined impact of rapid human population growth and climate change has been described as a ‘perfect storm’ that threatens our food security (Solomon *et al*., 2009; Godfray *et al*., 2010; Porter *et al*., 2014). Future predicted decreases in water availability and increased frequency of extreme drought and high‐temperature events are likely to present particular challenges for farmers, resulting in substantial crop losses (Vikram *et al*., 2015; Korres *et al*., 2017). Rice (*Oryza sativa*) is a major food crop, eaten by billions (Elert, 2014), and to mitigate the threat to global food security there is interest in developing new varieties of rice engineered to be ‘climate ready’.

Rice cultivation is particularly water intensive, using an estimated 2500 l of water per 1 kg of rice produced (Bouman, 2009). However, almost half of the global rice crop derives from rain‐fed agricultural systems where incidences of drought and high temperatures are predicted to become more frequent and damaging under climate change (Vikram *et al*., 2015; Matsuda *et al*., 2016; Korres *et al*., 2017). Like most land plants, rice uses microscopic pores called stomata to regulate CO_2_ uptake for photosynthesis with the concomitant release of water vapour via transpiration (Zeiger *et al*., 1987; Murchie *et al*., 2002). When water is plentiful, stomatal opening also permits regulation of plant temperature by evaporative cooling (Urban *et al*., 2017). Under water‐limiting drought conditions, stomatal closure slows down water loss, with potential trade‐offs being reduced carbon assimilation *A* and increased plant temperature (Hu *et al*., 2006; Tombesi *et al*., 2015; Urban *et al*., 2017). Elevated atmospheric CO_2_ concentrations also induce stomatal closure and raise plant temperature (Kollist *et al*., 2014; Engineer *et al*., 2016), but this response is typically not as important under field conditions as drought‐induced stomatal closure (Xu *et al*., 2016). In predicted future higher CO_2_ climates, it has been suggested that plants will be more water‐use efficient as enhanced photosynthetic *A* allows stomata to be less open, meaning less water will be lost (Keenan *et al*., 2013). However, despite grain yields increasing in experiments where rice is grown at elevated CO_2_, a greater volume of water is used than at current CO_2_ levels, indicating that, in the future, rice cultivation may be even more water intensive than it is today (Kumar *et al*., 2017).

Rising CO_2_ levels are expected to result in a warming of 1–4°C in global atmospheric temperatures by the end of the century, and the frequency of heat spikes will also increase (Meyer *et al*., 2014). Such dramatic rises in temperature are expected to lead to negative impacts on rice yields even in the presence of increased atmospheric CO_2_ (Ainsworth, 2008; Kumar *et al*., 2017). Rice is particularly sensitive to heat stress, with the majority of growth stages being affected once temperatures exceed 35°C (Redfern *et al*., 2012). This is especially the case during the reproductive period, (Redfern *et al*., 2012; Jagadish *et al*., 2015), and it is predicted that, by 2050, 27% of rice‐growing areas will experience at least 5 d of heat stress temperatures during this stage (Gourdji *et al*., 2013). The impact of heat stress is expected to be exacerbated as water resources diminish and more water‐use‐efficient practices involving less water are adopted. This may be somewhat mitigated if transpiration‐mediated cooling can be maintained, as rice can remain productive in air temperatures of 40°C if humidity remains low (Jagadish *et al*., 2015).

In addition to the reversible modification of stomatal apertures, plants in the longer term can adapt their stomatal development to optimize their stomatal conductance *g*_s_ to the surrounding environmental conditions, such as light intensity or CO_2_ concentration (Casson & Gray, 2008). At high temperature, some plant species can produce leaves with altered stomatal density, which can affect transpiration rates and evaporative cooling (Crawford *et al*., 2012; Jumrani *et al*., 2017). Currently, however, it is not known whether rice stomatal development is affected by growth temperature. In our study, we have investigated this and the feasibility of creating rice plants that require less water through genetically reducing stomatal density and *g*_s_. Our results indicate that in a future world with elevated atmospheric CO_2_, higher temperature and reduced water availability, stomatal‐based water conservation could help to maintain or even improve rice productivity by enhancing water conservation before drought and slowing water loss during drought.

Manipulating the number of stomata that form in plants requires detailed knowledge of the developmental programme. The regulation of stomatal function and development is well studied in the model dicot *Arabidopsis thaliana*, and recently researchers have begun to translate these findings into monocots, including some cereal crop species (Liu *et al*., 2009; Hughes *et al*., 2017; Raissig *et al*., 2017). During Arabidopsis epidermal development, the extracellular EPIDERMAL PATTERNING FACTOR (EPF) and EPF‐LIKE (EPFL) signalling peptides maintain the correct density and spacing of stomatal precursor cells through binding ERECTA‐family receptors (Hara *et al*., 2007, 2009; Hunt & Gray, 2009; Lee *et al*., 2015). Negative regulators of stomatal development, EPF2 and EPF1, restrict stomatal development. EPF2 primarily regulates asymmetric divisions which facilitate ‘entry’ to the stomatal lineage by forming meristemoids in the early epidermis, and EPF1 acts slightly later, to regulate stomatal spacing and the transition to a guard mother cell (GMC). EPFL9 (also known as STOMAGEN) competes with EPF2 for receptor binding and thus promotes stomatal development (Lee *et al*., 2015; Zoulias *et al*., 2018). Recently, it has been shown that epidermal patterning factors also regulate stomatal development in grasses (Hughes *et al*., 2017; Yin *et al*., 2017). As in Arabidopsis, there appear to be two *EPF* gene homologues that may restrict stomatal development in diploid grasses, but unlike Arabidopsis there are also two putative *EPFL9* genes (rather than one) (Hepworth *et al*., 2018). The combination of EPF/Ls required, and when they function during stomatal development in grasses, is not yet understood. Given that grass stomata develop in parallel files and have subsidiary cells (Stebbins & Shah, 1960), whereas dicot stomata typically develop in a more random pattern, it is probable that the factors regulating grass stomatal development have evolved additional and or modified functions to their Arabidopsis/dicot counterparts (Facette & Smith, 2012; Raissig *et al*., 2016). So far, one rice and one barley (*Hordeum vulgare*) epidermal patterning factor have been shown to affect stomatal development in grasses (Hughes *et al*., 2017; Yin *et al*., 2017). In rice, lack of *OsEPFL9a* expression results in reduced stomatal density (Yin *et al*., 2017), and overexpression of *HvEPF1* in barley leads to reduced stomatal density, with *HvEPF1* appearing to act both before and the after the asymmetric ‘entry’ division; that is, HvEPF1 has functional attributes reminiscent of both Arabidopsis EPF1 and EPF2 activities (Hughes *et al*., 2017). By reducing stomatal density, Hughes *et al*. improved barley drought tolerance, but did not quantify reductions in water use, nor investigate how fewer stomata impacted on growth at high temperature or elevated atmospheric CO_2_ concentrations. Here, we investigate how reducing stomatal density in the major food crop, rice, affects water use, drought tolerance and heat stress tolerance, in experiments carried out at atmospheric CO_2_ levels expected to be prevalent in the field over the next 20–50 years (Solomon *et al*., 2009; Meyer *et al*., 2014).

## Materials and Methods

### Plant growth conditions

Rice cultivar ‘IR64’ (*Oryza sativa* L. ssp*. indica*) seeds were germinated and seedlings cultivated for 7–8 d in a Petri dish with 15 ml water in a Sanyo growth cabinet with a 12 h 26°C : 12 h 24°C light : dark cycle, photosynthetically active radiation (PAR) 200 μmol m^−2^ s^−1^. Seedlings were transferred to 13D pots (0.88 l), or for yield experiments to large 19F pots (2.4 l) (East Riding Horticulture, York, UK) containing soil consisting of 71% Kettering Loam (Boughton, UK), 23.5% Vitax John Innes No. 3 (Leicester, UK), 5% silica sand and 0.5% Osmocote Extract Standard 5–6 month slow‐release fertilizer (ICL, Ipswich, UK) by volume saturated with water. Plants were grown in Conviron controlled‐environment growth cabinets (Controlled Environments Ltd, Winnipeg, MB, Canada) at 12 h 30°C : 12 h 24°C light : dark cycle, PAR 1000 μmol m^−2^ s^−1^ and 60% relative humidity, with a constant supply of water to the pot base and watering from the top once a week unless otherwise stated. Plants were propagated at an atmospheric CO_2_ concentration of 450–480 ppm maintained by a pressurized CO_2_ tank (BOC) to the ambient conditions of the growth chamber when required. For higher temperature experiments, the daytime temperature was raised to 35 or 40°C and humidity adjusted to maintain 60% relative humidity.

For yield experiments, plants were fertilized every 14 d from 42 d with 0.5 g l^−1^ Chempak High Nitrogen Feed No. 2, except when water was withheld (Thompson & Morgan, Ipswich, UK). Treatment 1 plants, which were well watered throughout the experiment, were harvested after 105 d. Treatment 2, which were droughted twice during vegetative growth at 28 d (for 9 d) and at 56 d (for 7 d), were harvested after 120 d. Treatment 3 plants, which were droughted during flowering at 88 d (for 3 d), were harvested after 126 d. Plant tissue was dried at room temperature for 1 month for yield analysis. Flowering of plants in treatments 1 and 3 began at *c*. 80 d after germination. For treatment 2 this occurred after *c*. 92 d. There was no obvious difference in flowering time between genotypes. For treatment 1, *n *=* *8; for treatment 2, *n *=* *5–7; and for treatment 3, *n *=* *6–7. Stomatal densities of leaf abaxial surfaces were recorded from all plants across all treatments and experiments. Impressions of leaf 5 were taken after infrared gas exchange analysis had been carried out. Cumulative water loss was assessed by growing each plant in an individual tray filled with 750 ml of water; this was then weighed daily and water subsequently replenished to 750 ml. Soil‐filled pots, with or without plants, were placed in randomized positions within the growth chamber, and weighed and returned to different positions every day. The mean value of water evaporated from control pots without plants was subtracted from each experimental value to determine water loss from each plant.

Arabidopsis plants were grown on M3 Levington compost in Conviron growth chambers with 9 h 22°C : 15 h 16°C, light : dark cycle, PAR 200 μmol m^−2^ s^−1^, 65% humidity and 450–480 ppm CO_2_.

### Generation of transgenic lines

The overexpression construct was made by PCR amplifying the rice *OsEPF1* cDNA (*OSIR64_00232g011350.1*) (F: CACCATGAGGAGGCACGCTACTC; R: CTAGCTGGAGGGCACAGGGTA) and inserting into the pENTR/D‐TOPO vector (Thermo Fisher, Waltham, MA, USA). An LR Clonase™ reaction (Thermo Fisher, Waltham, MA, USA) was used to transfer the *OsEPF1* coding sequence into pSC310 vector used for rice transformations. ‘IR64’ plants were transformed as previously described (Yin *et al*., 2017). Control ‘IR64’ plants had been through the same tissue culture and regeneration process, but did not contain a transgene. Plants from the second or third (T_2_ or T_3_) generations after regeneration were used for collection of experimental data. For Arabidopsis overexpression experiments, *OsEPF1* cDNA was transferred from pENTR/D‐TOPO vector to pMDC32 via an LR Clonase reaction. For *AtEPF2* promoter fusion to *OsEPF1*, pMDC99 was digested using *Kpn*I and then blunt ends generated using DNA Polymerase I. The *AtEPF2* promoter (Hunt & Gray, 2009) was amplified and ligated into pMDC99. The *pMDC99::AtEPF2pro* plasmid was digested with *Asc*I and *Pac*I and a PCR‐amplified sequence (F: CGCGCCATGAGGAGGCACGCTACT; R: ATTAACTAGCTGGAGGGCACAGGG) was ligated downstream of the *AtEPF2* promoter. Arabidopsis transformations were performed as previously described. The Arabidopsis *epf2* knockout was used for complementation experiments (Hunt & Gray, 2009). Successful transformation was confirmed by PCR of plant genomic DNA and cellular measurements.

### Southern blots

Genomic DNA extracted from T_0_ plants was restricted with *Eco*RI, separated by electrophoresis, blotted onto a membrane and probed with the maize (*Zea mays*) ubiquitin promoter sequence amplified and labelled using primers (F: TCTAACGGACACCAACCAGC; R: GAGGTTGGGGAAAGAGGGTG) as described (Yin *et al*., 2017).

### Analysis of *OsEPF* transcript levels

RNA from whole 8‐d‐old rice seedlings was extracted using Spectrum™ Plant Total RNA Kit (Sigma‐Aldrich, Gillingham, UK), adjusted to 100 ng μl^−1^ per sample, treated with DNA‐free™ DNA Removal Kit (Thermo Fisher Scientific, Waltham, MA, USA) and converted to cDNA using M‐MLV Reverse Transcriptase (200 U μl^−1^) (Thermo Fisher Scientific). Real‐time quantitative PCR analysis was performed using the Rotor‐Gene SYBR Green PCR Kit (400) and a Corbett Rotor Gene 6000 (Qiagen) using primers (F: CCCCTTTTCCACAGATGATGTAGTA; R: GCTGTGGCCTGTGGTGAGA). Relative expression values were calculated by normalizing the take‐off value and amplification efficiency of the genes analysed relative to the Profilin (LOC_Os06g05880) housekeeping gene (van Campen *et al*., 2016).

### Epidermal imaging, quantification and calculation of *g*_s max_

**Figure 1:**
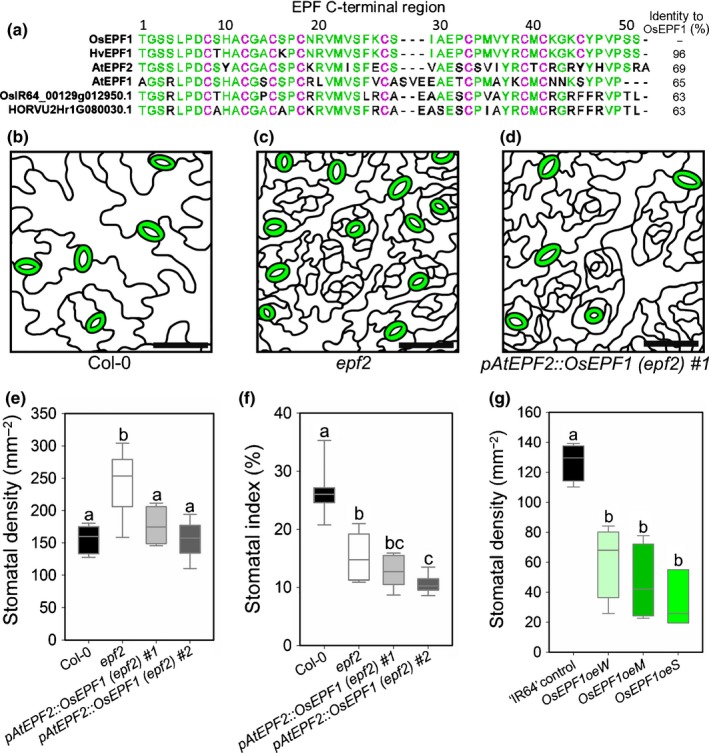
The rice EPIDERMAL PATTERNING FACTOR OsEPF1 (OSIR64_00232g011350.1) negatively regulates stomatal development in *Arabidopsis thaliana* and the rice cultivar ‘IR64’ (*Oryza sativa* ssp*. indica*). (a) Peptide sequence alignment of the C‐terminal region of closely related EPFs in rice, barley (*Hordeum vulgare*) and Arabidopsis. Rice OsEPF1, like barley HvEPF1, has nine cysteine residues (purple) in the C‐terminal region. Additional amino acid residues identical to OsEPF1 are marked green. Percentage sequence identity of other EPF peptides to OsEPF1 shown on the right. HvEPF1, HORVU2Hr1G116010.3; AtEPF2, AT1G34245.1; AtEPF1, AT2G20875.1. (b–d) Tracing of images of the mature abaxial epidermis of 56‐d‐old Arabidopsis leaves. (b) Arabidopsis Col‐0 background ecotype, (c) *epf2* and (d) *pAtEPF2::OsEPF1* (*epf2*) #1 (bars, 50 μm). (e) Stomatal density and (f) stomatal index of Col‐0, *epf2* and two independent complemented lines: *pAtEPF2::OsEPF1* (*epf2*) *#1* and *#2*. (g) Stomatal density of the first true leaf of three independent T_2_ generation *OsEPF1* overexpressing ‘IR64’ rice lines: *OsEPF1oeW* (weak), *OsEPF1oeM* (medium) and *OsEPF1oeS* (strong phenotype) at the 8‐d‐old seedling stage. For graphs (e–g), horizontal lines within boxes indicate the median and boxes indicate the upper (75%) and lower (25%) quartiles. Whiskers indicate the ranges of the minimum and maximum values, and different letters indicate a significant difference between the means (*P *<* *0.05, one‐way ANOVA). (e, f) *n *=* *7 plants; (g) *n *=* *4 plants.

Cell densities and tracings of Arabidopsis epidermis were produced in Paint.net (https://www.getpaint.net/) from nail varnish peels of dental resin impressions of fully expanded leaves from 63‐d‐old plants. For Fig. 1 (8‐d‐old rice seedlings, leaf 1) and Fig. 5 (21‐d‐old rice plants, leaf 5), epidermal cell densities were also recorded from nail varnish peels of dental resin impressions, calculating averages at six veins in from the leaf edge, from four 0.147 mm^2^ fields of view per replicate. Images were taken on an Olympus BX51 microscope with an Olympus DP71 camera. The leaf 5 values of stomatal density *D* (mm^−2^) were used in in Eqn 1. Epidermal cell densities were calculated using four 350 μm^2^ confocal stacks per replicate, taken on a Nikon A1.

**Figure 2:**
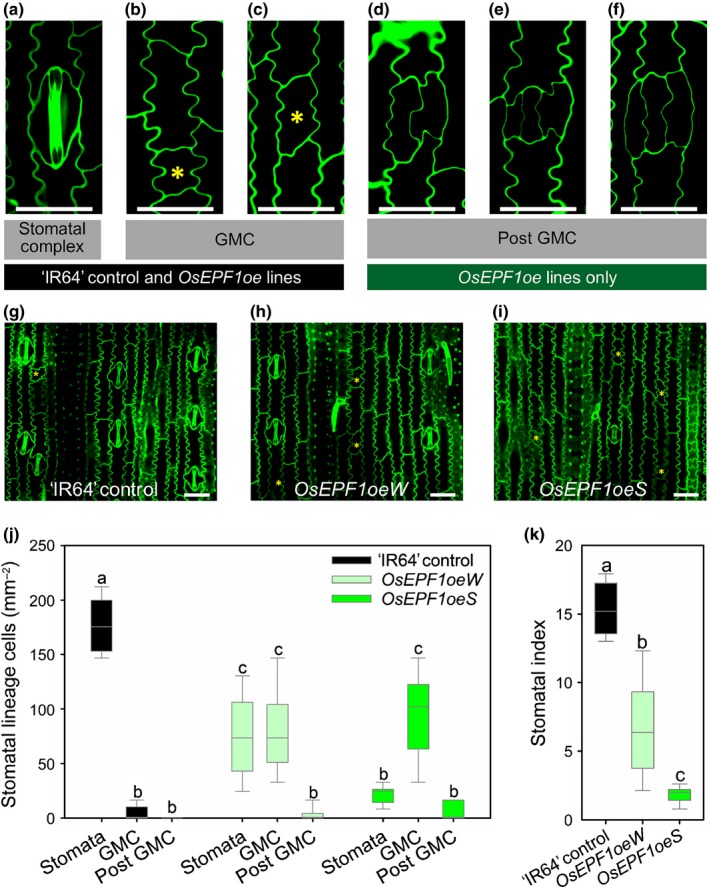
Overexpressing *OsEPF1* restricts stomatal development in ‘IR64’ rice (*Oryza sativa* ssp*. indica*). Confocal images of the abaxial epidermis of the fifth fully expanded true leaf of ‘IR64’ control, *OsEPF1oeW* and *OsEPF1oeS* plants showing interdigitating pavement cells surrounding (a) a stomatal complex comprised of two outer subsidiary cells and two inner guard cells and (b–f) arrested stomatal lineage cells, comprising (b, c) guard mother cells (GMCs; yellow asterisks) and (d–f) post‐GMC arrested cells. Epidermal images of (g) ‘IR64’, (h) *OsEPF1oeW* and (i) *OsEPF1oeS* lines. Yellow asterisks denote GMCs. Bars, 25 μm. (j) Stomatal lineage cell density and (k) stomatal index. For graphs (j, k), horizontal lines within boxes indicate the median, and boxes indicate the upper (75%) and lower (25%) quartiles. Whiskers indicate the ranges of the minimum and maximum values, and different letters indicate values with a significantly different mean within graphs (*P *<* *0.05, one‐way ANOVA). *n *=* *6 plants.

Stomatal density and stomatal index in Figs 1, 2 and 5 and Supporting Information Fig. S1 were measured using the cell counter plugin of ImageJ (Fiji v.1.51u). Complex size (Fig. S2) was manually measured in ImageJ from a total of 30 stomata from each genotype, taken from six biological replicates (five complexes per plant). The images in Figs 2 and S2 were taken as previously described (Hughes *et al*., 2017). For calculating pore aperture, guard cell area and *g*_s max_ (Figs 5, S3), 20 stomata per plant (five per field of view) from six (40°C) or seven (30°C) biological replicates were imaged. Pore area was calculated as an ellipse from the major axis of measured aperture length, and the minor axis measured aperture width at the centre of the pore. Guard cell area was calculated as an ellipse from the axes of measured guard cell length and the doubled guard cell width at the centre of the stoma. Maximum pore aperture *a*_max_ (μm^2^) was calculated as an ellipse from axes equal to the measured aperture length and half of the aperture length. Pore depth *l* (μm) was taken as equal to guard cell width at the centre of the stoma. Abaxial anatomical *g*_s max_ was calculated using the double end‐corrected version of the Franks & Farquhar (2001) equation, from Dow *et al*. (2014):

$$\text{Abaxial anatomical}\;gs_{\max}=\left(d\cdot D\cdot a_{\max}\right)/\left(v\cdot \left(l+\left(\pi /2\right)\cdot \surd \left(a_{\max}/\pi \right)\right)\right)$$

where *d* (m^2^ s^−1^) is the diffusivity of water in air and *v* (m^3^ mol^−1^) is the molar volume of air. Assuming equal stomatal densities on both sides of the leaf, this value was doubled to give total anatomical *g*_s max_. Values used in calculations are shown in Table S1.

### Physiological measurements

**Figure 3:**
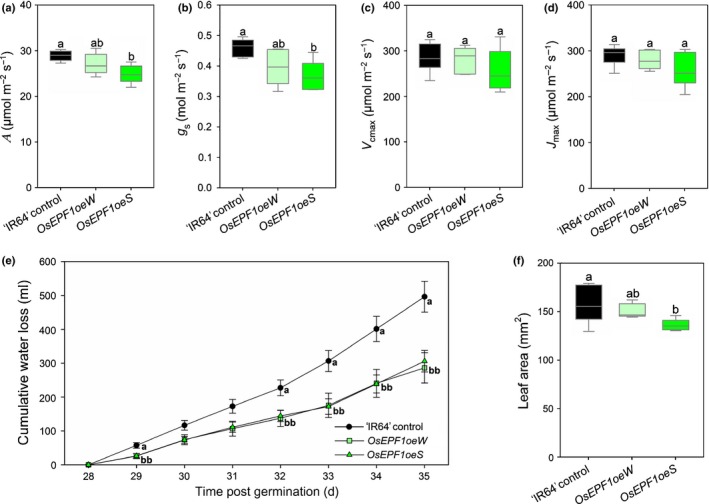
Plant gas exchange and water loss in ‘IR64’ control and *OsEPF1oe* rice (*Oryza sativa* ssp*. indica*). (a) Infrared gas exchange analysis of carbon assimilation *A* and (b) stomatal conductance *g*_s_ performed at a light intensity of 1000 μmol m^−2 ^s^−1^ photosynthetically active radiation (PAR) akin to growth‐chamber conditions. (c) Maximum velocity of Rubisco *V*_cmax_ and (d) the potential rate of electron transport *J*_max_ of plants grown under saturating light conditions (2000 μmol m^−2^ s^−1^ PAR). For (a–d) measurements were performed on the fifth fully expanded true leaf of 21‐d‐old plants. (e) Cumulative weight loss over 7 d without watering, starting from 28 d post germination. (f) Total leaf area of plants 28 d post germination. For graphs (a–d, f), horizontal lines within boxes indicate the median, and boxes indicate the upper (75%) and lower (25%) quartiles. Whiskers indicate the ranges of the minimum and maximum values and different letters indicate values with a significantly different mean within graph (*P *<* *0.05, one‐way ANOVA). Error bars in (e) indicate SEM. (a–d) *n *=* *6 plants; (e) *n *=* *10 plants; (f) *n *=* *5 plants.

Gas exchange measurements were performed on 21‐d‐old plants on fully expanded true leaf 5. Measurements for Figs 3(a,b) and S4(a) were taken on a LiCOR 6400 infrared gas analyser (Lincoln, NE, USA). The leaf chamber conditions were: light intensity 1000 μmol m^−2^ s^−1^ PAR, humidity 60%, leaf temperature 30°C, flow 300 μmol s^−1^ and CO_2_ concentration 480 ppm. For Figs 3(c,d), 5(d–g) and S4(b,c), a Li‐Cor 6800 infrared gas analyser (Lincoln, NE, USA) was used. Light curve analysis was conducted using the same chamber conditions with 3–5 min stabilization between each light level. The light levels were 2000, 1500, 1200, 1000, 800, 600, 480, 340, 200, 100 and 50 μmol m^−2^ s^−1^ PAR. CO_2_ response curves were taken under saturating light (2000 m^−2^ s^−1^ PAR) starting at 480 ppm CO_2_ concentration and then lowered to 340, 200, 150, 125, 100, 75, 50, 25 and finally 0 ppm CO_2_. Plants were re‐acclimatized at 480 ppm CO_2_ and then CO_2_ was raised to 600, 800, 1000, 1250 and 1500 ppm. For all steps, plants were allowed 2.5–5 min stabilization time. Values of maximum rate of Rubisco carboxylase activity *V*_cmax_ and potential rate of electron transport *J*_max_ were calculated using the Excel tool from Sharkey *et al*. (2007). For plants grown at different temperatures (30, 35 or 40°C), leaf chamber temperature was set equivalent to growth temperature with light set to 2000 μmol m^−2^ s^−1^ PAR and other conditions were as already noted. *F*_v_/*F*_m_ values were measured 1 h before onset of photoperiod, with a FluorPen FP 100 (PSI, Drasov, Czech Republic). Thermal images were captured using an FLIR T650sc, and quantification of temperature was performed using FLIR Tools (www.flir.co.uk). Data were collected from equivalent areas of mature leaves across treatments.

### Leaf area analysis

Total leaf area was measured from five 28‐d‐old plants per genotype, by excising every leaf where it emerged from the sheath, flattening and imaging. Areas were calculated in ImageJ (Fiji v.1.51u) using thresholding and the magic wand tool.

### Amino acid sequence alignments

The Arabidopsis EPF2 peptide sequence was used in Blast searches for EPF peptides sequences in ‘IR64’ rice via the Rice SNP‐Seek database (http://snp-seek.irri.org/_locus.zul;jsessionid=096476AC6709F1EED57798F6D6756EE0) (Alexandrov *et al*., 2015). Arabidopsis and barley sequences were obtained from Phytozome v.12.1.6 (Goodstein *et al*., 2012) and aligned using Muscle using defaults setting on Jalview v.2 (Edgar, 2004; Waterhouse *et al*., 2009).

### Graphs and statistical analysis

Graphs were produced and statistical analysis conducted using Sigmaplot v.13 (Systat Software, Inc., San Jose, CA, USA) and one‐way ANOVA, except in Figs 5(b) and S3, where two‐way ANOVAs were performed. If unequal variances were detected in ANOVAs, a Kruskal–Wallis one‐way ANOVA on ranks was performed.

## Results

In Arabidopsis and barley, *AtEPF2* or *HvEPF1* overexpression reduces stomatal density, leading to improved drought tolerance (Hara *et al*., 2009; Hunt & Gray, 2009; Franks *et al*., 2015; Hepworth *et al*., 2015; Hughes *et al*., 2017). Two closely related rice gene products have been identified as orthologues of Arabidopsis *EPF1* and *EPF2*, both potentially involved in regulating stomatal development (Hepworth *et al*., 2018) (Fig. 1a). In the ‘IR64’ rice cultivar genome, *OSIR64_00232g011350.1* encodes the most similar gene product to AtEPF2 and HvEPF1. We studied the function of this rice gene product by fusing the *OSIR64_00232g011350.1* coding sequence to the native *AtEPF2* promoter and expressing the gene construct in the Arabidopsis *epf2* knockout background (Figs 1, S1). Expression of this ‘*OsEPF* rescue’ gene construct restored the Arabidopsis *epf2* stomatal density from *c*. 250 mm^−2^ back to normal levels (*c*. 160 mm^−2^) (Fig. 1b–e). However, high numbers of aborted stomatal lineage cells, characteristic of *epf2,* persisted in the epidermis, suggesting that in Arabidopsis plants the expression of the ‘*OsEPF* rescue’ gene could not adequately restrict the number of asymmetric ‘entry’ divisions at the start of the stomatal development pathway (Figs 1b–d, S1). Excessive stomatal lineage cells formed but were unable to progress to stomata; this phenotype was previously observed in Arabidopsis *EPF1* overexpression experiments (Hara *et al*., 2009). Owing to the large number of aborted stomatal lineage cells, the stomatal indices (ratio of stomata to stomata plus other epidermal cells) of the *OsEPF* rescue plants remained similar to *epf2* plants (Fig. 1f). Ectopic overexpression of *OSIR64_00232g011350.1* in Arabidopsis, directed by the CaMV35S promoter, led to a marked reduction in both stomatal density and stomatal index (Fig. S1). Based on these analyses of OSIR64_00232g011350.1 function in Arabidopsis stomatal development, and the similarities to *HvEPF1* overexpression in Arabidopsis (Hughes *et al*., 2017), we designate *OSIR64_00232g011350* as *OsEPF1*.

We engineered the ‘IR64’ rice cultivar to ectopically overexpress *OsEPF1* under the control of the maize ubiquitin promotor. Analysis of the first true leaf from T_2_ generation seedlings from three independently transformed rice lines identified a range of reduced stomatal density phenotypes that we classified as weak, moderate or strong (W, M or S, with seedling stomatal densities of 62 mm^−2^, 46 mm^−2^ and 33 mm^−2^ respectively, in comparison with ‘IR64’ at 127 mm^−2^; Fig. 1g). Southern blot analysis suggested a single copy of the transgene in *OsEPF1oeW*, three copies in *OsEPF1oeM* and six copies in *OsEPF1oeS* (Fig. S5). Quantitative reverse transcription PCR (RT‐qPCR) confirmed *OsEPF1* overexpression in all lines, with *OsEPF1oeS* exhibiting the highest level of expression (Fig. S5). The *OsEPF1oeW* and ‐*S* lines were used in all subsequent experiments, which were carried out on plants grown at an elevated 450–480 ppm CO_2_ concentration to simulate the elevated atmospheric CO_2_ level that we are expected to have reached by the middle of this century (Solomon *et al*., 2009).

The mature rice leaf epidermis of ‘IR64’ control plants normally contains interdigitating pavement cells, stomatal complexes made up of guard cells and subsidiary cells, and occasionally, arrested stomatal precursor cells known as GMCs (Fig. 2a–c). Detailed analysis of the fully expanded fifth mature rice leaf of *OsEPF1oe* lines revealed increased incidences of arrested GMCs, and unusually some instances of post‐GMC cells that had also failed to develop into mature stomatal complexes (Fig. 2d–f). Stomatal density was reduced by 58% for *OsEPF1oeW* and 88% for *OsEPF1oeS* relative to ‘IR64’ controls (Fig. 2g–j). The reduced capacity of *OsEPF1oe* to produce mature stomatal complexes also led to reduced stomatal indices (Fig. 2k), indicating that, as observed in Arabidopsis (Fig. S1), *OsEPF1* inhibits both stomatal initiation and stomatal lineage progression in rice when ectopically overexpressed. Observation of subepidermal layers of *OsEPF1oe* leaves confirmed that substomatal cavities only formed in association with mature stomatal complexes and did not form beneath arrested precursor cells (Fig. S2). In addition, *OsEPF1oeS* stomatal complexes were found to be 12% smaller than ‘IR64’ controls, and there was a small increase (*P *<* *0.05) in *OsEPF1oeS* vein density, but leaf width and the number of veins across the width of the leaf were not significantly altered (Fig. S2).

We performed infrared gas exchange analysis to determine whether reduced stomatal density and size led to reductions in *A* and/or stomatal conductance *g*_s_ and to assess whether changes had arisen in plant photochemistry (Figs 3a–d, S4). Grown at 450–480 ppm CO_2_, *OsEPF1oeW* steady‐state *A* and *g*_s_ were similar to ‘IR64’ controls; but in the more severe *OsEPF1oeS* line, reductions in both *A* and *g*_s_ were observed (*P *<* *0.05; Fig. 3a,b). We measured gas exchange across a range of light intensities and in this experiment found no significant differences in *A* between genotypes at and below the growth light intensity (1000 μmol m^−2^ s^−1^ PAR; Fig. S4). However, above this light intensity, *A* was reduced relative to the ‘IR64’ controls in both *OsEPF1oe* lines. To assess whether the maximum rate of Rubisco carboxylase activity *V*_cmax_ or the potential rate of electron transport *J*_max_ was altered in plants with reduced stomatal density, we measured *A* and intercellular CO_2_ at a range of CO_2_ concentrations (Figs 3c,d, S4). We did not detect any significant differences in the rates of either *V*_cmax_ or *J*_max_, suggesting that the photosynthetic apparatus in *OsEPF1oe* plants can perform at equivalent rates to controls. To see whether changes in stomatal density and gas exchange properties reduced whole‐plant water use, we directly measured water loss between weeks 4 and 5 (Fig. 3e). Over this 1 wk period, both *OsEPF1oe* lines used significantly less water than ‘IR64’ controls did, with *OsEPF1oeW* using 42% less water and *OsEPF1oeS* using 38% less water. To determine whether the observed reduction in water loss could be affected by plant size, we measured whole plant leaf area on a subset of 4‐wk‐old plants and found that *OsEPF1oeW* plants had no reduction in size (*P *=* *0.33), but *OsEPF1oeS* had a 14% reduction in leaf area (*P *=* *0.04; Fig. 3f).

**Figure 4:**
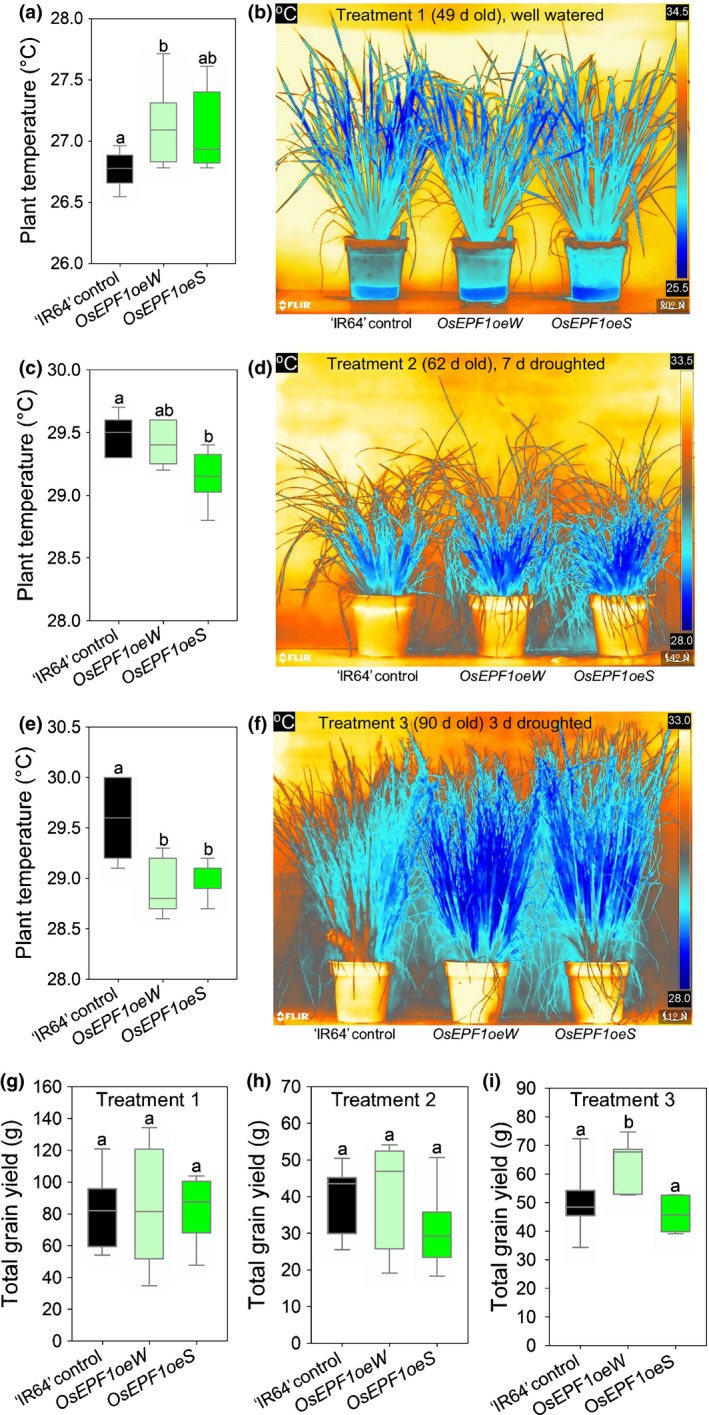
*OsEPF1* overexpression affects leaf water loss and temperature, and enhances yield following flowering drought in ‘IR64’ rice (*Oryza sativa* ssp*. indica*). (a, b, g) Treatment 1: well‐watered plants. (c, d, h) Treatment 2: water withheld during vegetative growth at 28 d for 9 d and at 56 d for 7 d. (e, f, i) Treatment 3: water withheld during reproductive stage at 88 d for 3 d. Surface temperatures of (a) treatment 1 plants, well‐watered at 49 d old, (c) treatment 2 plants, 62 d old at the end of 7 d drought period, and (e) treatment 3 plants 90 d old at the end of 3 d drought period. Infrared thermal images in (b), (d) and (f) are from representative plants used to compile data in (a), (c) and (e). Dark blue denotes coolest areas, as indicated on scale on right. (g–i) Total grain yields of (g) well‐watered, (h) vegetative drought and (i) flowering drought plants. For all box plots graphs, horizontal lines within boxes indicate the median with boxes covering the upper (75%) and lower (25%) quartiles. Whiskers indicate the ranges of the minimum and maximum values, and letters indicate significantly different mean values (*P *<* *0.05, one‐way ANOVA). Owing to unequal variances, in (g) a Kruskal–Wallis one‐way ANOVA on ranks was performed: (a, b, g) *n *=* *8; (c, d, h) *n *=* *5–7; (e, f, j) *n *=* *6–7.

To test whether the substantial reductions in *OsEPF1oe* stomatal density could lead to improvements in drought tolerance, plants were grown in 2.4 l pots and subjected to one of three different watering regimes (Figs 4, S6, S7). Treatment 1 plants were watered normally; treatment 2 plants were subjected to two periods without water during vegetative growth at 28 d (for 9 d) and at 56 d (for 7 d); and treatment 3 plants were subjected to a single drought period (for 3 d) when plants were 88 d old and flowers had emerged from panicle sheaths.

We used infrared thermal imaging to assess how altering stomatal development affected evaporative cooling. In treatment 1 conditions, low stomatal density *OsEPF1oe* lines were *c*. 0.3°C warmer than ‘IR64’ controls at the maximum tillering stage (49 d old), suggesting a small but significant reduction in water loss and cooling (Fig. 4a,b). Conversely, when watering ceased during treatments 2 and 3, *OsEPF1oe* plants were cooler than ‘IR64’ controls (*OsEPF1oeS* were 0.3°C cooler towards the end of drought period during treatment 2; *OsEPF1oeW* and *OsEPF1oeS* were 0.7 and 0.6°C cooler during treatment 3; Figs 4c–f, S6). Thus, *OsEPF1oe* plants were able to maintain evaporative cooling at higher levels than controls during drought, suggesting that initial improved water conservation in the reduced stomatal density lines allowed plants to keep their stomata open for longer under drought conditions.

To investigate whether either the reduced *g*_s_ that we observed when plants were well watered or the enhanced evaporative cooling observed during vegetative and reproductive drought could affect plant growth or productivity, we grew the *OsEPF1oe* and control plants to maturity. After treatments 1 and 2, *OsEPF1oe* plant biomass and grain yield were equivalent to the ‘IR64’ control plants (Figs 4g,h, S7). Interestingly, following treatment 3, drought during the flowering period, the *OsEPF1oeW* line produced significantly more aboveground biomass (26% increase) and grain yield (27% increase) than ‘IR64’ controls did (*P *<* *0.01 and *P *<* *0.05), whilst *OsEPF1oeS* yields remained comparable to ‘IR64’ (Figs 4i, S7). The 1000 grain weight of both *OsEPF1oe* lines was also significantly higher than that of ‘IR64’ controls in treatment 3 (*P *<* *0.01), suggesting that having fewer stomata has a positive effect on grain filling when plants experience drought during flowering (Fig. S7).

**Figure 5:**
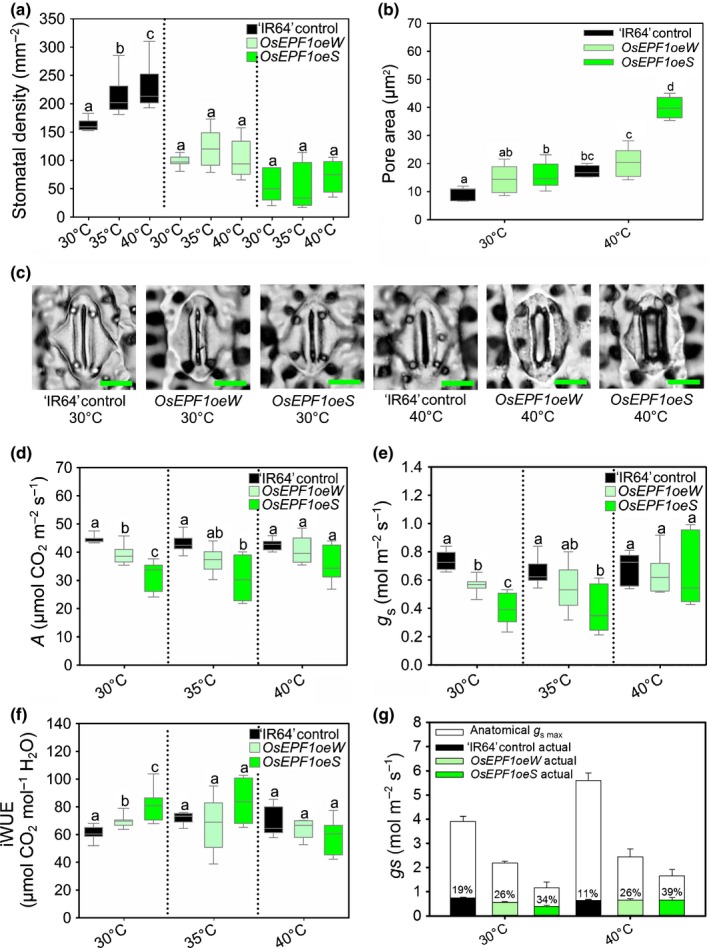
Stomatal development and physiological responses on the fully expanded true leaf 5 of ‘IR64’ control, *OsEPF1oeW* and *OsEPF1oeS* rice (*Oryza sativa* ssp*. indica*) grown at 30, 35 or 40°C. (a) Stomatal density, (b) calculated stomatal pore area at 30 and 40°C, (c) representative images of individual stomates at 30 and 40°C (bars, 10 μm), (d) carbon assimilation *A*, (e) stomatal conductance *g*_s_, (f) intrinsic water use efficiency (iWUE,* A/g*_s_) and (g) anatomical potential *g*_s max_ with actual *g*_s_ values plotted, showing the percentage of potential *g*_s_ that was reached. All infrared gas exchange analysis was performed at 2000 μmol m^−2 ^s^−1^ PAR. For graphs (a, b, d–f), horizontal lines within boxes indicate the median, and boxes indicate the upper (75%) and lower (25%) quartiles. Whiskers indicate the ranges of the minimum and maximum values. (a) A one‐way (ANOVA) statistical analysis was carried out to identify significant differences between temperatures within genotypes; for (b) a two‐way ANOVA was used, and for (d–f) one‐way ANOVA analyses were carried out to identify significant differences between genotypes within a given temperature treatment. Dotted lines separate the different groups for statistical analyses. Letters within a group indicate significantly different mean values (*P* < 0.05, one‐way ANOVA). Owing to unequal variance, in (d) a Kruskal–Wallis one‐way ANOVA on ranks was performed. *n* = 6–7 plants.

To examine whether *OsEPF1oe* plants have altered heat stress tolerance, a series of experiments was performed at elevated atmospheric CO_2_ and elevated daytime temperatures (35 or 40°C compared with a normal growth condition of 30°C) (Fig. 5). We noted that growth at higher temperatures affected stomatal development in rice controls: ‘IR64’ produced leaves with a 31% increase in stomatal density at 35°C and a 40% increase at 40°C. *OsEPF1oe* plants, however, were unable to adjust stomatal density across temperature treatments, suggesting that this developmental response may require modulation of EPF levels (Fig. 5a). To see whether changes in stomatal density were accompanied by anatomical changes to stomata at high temperature, we also measured guard cell size and stomatal pore area of plants grown at 30 and 40°C (Figs 5b,c, S3). At 30 and 40°C, *OsEPF1oeW* had similar‐sized guard cells to controls, whereas *OsEPF1oeS* guard cells were significantly smaller (30°C, *P < *0.001 and 40°C *P < *0.01*,* Fig. S3). For all plants, stomatal pore area was significantly increased at 40°C (*P < *0.05), with *OsEPF1oeS* plants having significantly larger pore areas than controls at 30 and 40°C (Fig. 5b,c). These data suggest that *OsEPF1oe* plants increased stomatal aperture (but not guard cell size) to compensate for reduced stomatal density, with this response being particularly noticeable at 40°C.

To see how *OsEPF1oe* plants might perform at high temperature with light levels similar to a bright sunny day in the field (Murchie *et al*., 2002), we conducted steady‐state infrared gas exchange analysis on plants grown at the 30, 35 and 40°C with leaf chamber light levels set to 2000 μmol m^−2^ s^−1^ PAR (Fig. 5d–f). At 30°C all *OsEPF1oe* plants had significantly lower *A* and *g*_s_ than ‘IR64’ controls did (*P < *0.05; Fig. 5d). However, when *OsEPF1oeW* plants were grown at 35°C, *A* and *g*_s_ were both comparable to controls; and when grown at 40°C, neither of the *OsEPF1oe* lines differed significantly from ‘IR64’ plants in these parameters (Fig. 5d,e). Calculation of intrinsic water use efficiency *A*/*g*_s_ (iWUE) showed that at 30°C *OsEPF1oe* plants performed significantly better than ‘IR64’ controls (*P *<* *0.05); at 35 or 40°C this was not the case, and *OsEPF1oe* iWUE levels were similar to controls (Fig. 5f). The failure of *OsEPF1oe* plants to maintain improved iWUE at higher temperatures may be explained by the increase in the *g*_s_ of *OsEPF1oe* to a level similar to that of control plants (Fig. 5d). Taken together with our finding that *OsEPF1* plants had larger apertures when grown at 40°C (Fig. 5b,c), our data suggest that plants with reduced stomatal density can compensate for having fewer stomata by increasing stomatal aperture when assayed under high temperature and high light intensity.

To estimate the physical limitations associated with having a reduced stomatal density we calculated maximum stomatal conductance *g*_s max_ using the formula set out in Dow *et al*. (2014) (Fig. 5g; Table S1). By comparing the calculated potential *g*_s m*a*x_ with the actual *g*_s_ values measured at 2000 μmol m^−2^ s^−1^ PAR, it can be seen that at 30 and 40°C the *OsEPF1oeS* plants are operating at over a third of their maximum capacity whereas controls operate at below 20% capacity. These data suggest that there are clearly opportunities to reduce stomatal density whilst maintaining *g*_s_ in the hot conditions expected to become more prevalent in the coming decades.

**Figure 6:**
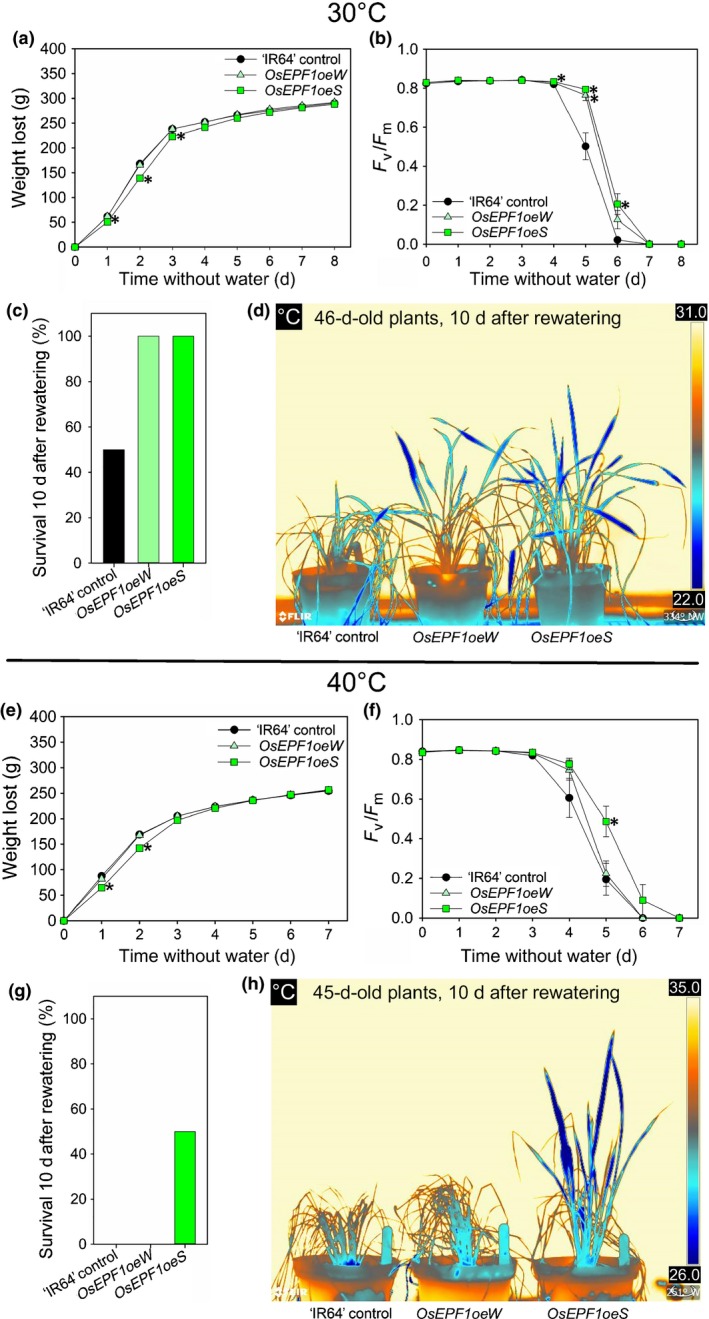
Increased survival rate of *OsEPF1oe* plants following severe drought at 30 or 40°C in ‘IR64’ rice (*Oryza sativa* ssp*. indica*). (a, e) Cumulative water loss over drought period imposed on 28‐d‐old ‘IR64’ control, *OsEPF1oeW* and *OsEPF1oeS* plants grown at (a) 30°C or (e) 40°C in 0.88 l pots. Dark‐adapted *F*_v_/*F*_m_ over drought period at (b) 30°C or (f) 40°C. Percentage of plants surviving 10 d after rewatering following (c) 8 d (30°C) or (g) 7 d (40°C) of total water withdrawal. Thermal images of plants 10 d after rewatering grown at (d) 30°C or (h) 40°C. Dark blue represents the coolest areas, as shown on scales on right. One‐way ANOVAs were performed to compare values for each day in each of the experiments conducted in (a, b, e, f). Asterisks indicate *P *<* *0.05 significance groups. *n *=* *10 plants. Error bars are plus/minus SEM. Owing to unequal variance, in (d) a Kruskal–Wallis one‐way ANOVA on ranks was performed.

Having measured the performance of the *OsEPF1oe* reduced stomatal density plants under different drought or heat treatments, we investigated the combined effects of both these abiotic stresses (Fig. 6). Before imposing the drought treatment, we measured temperatures of plants grown at 40°C and observed no differences between *OsEPF1oe* plants and controls, indicating that reducing stomatal density did not cause overheating under these conditions (Fig. S8). From 28 d post germination we imposed severe drought at either 30°C (for 8 d) or 40°C (for 7 d) during the vegetative growth period. During the water withdrawal period, *OsEPF1oeW* plants lost water (indicated by a reduction in pot weight) at a similar rate to control plants, whereas *OsEPF1oeS* plants showed significantly increased water conservation at both temperatures (for 3 d at 30°C, *P *<* *0.05, and 2 d at 40°C, *P *<* *0.001; Fig. 6a,e). As when grown at 30°C, we noticed that *OsEPF1oeS* plants appeared smaller when grown at 40°C, so we assessed tiller development at 5 wk post germination and found that, although not significantly different (*P = *0.057), *OsEPF1oeS* showed a trend towards reduced tiller number (Fig. S8). Analysis of dark‐adapted *F*_v_/*F*_m_ chlorophyll fluorescence values (an indicator of abiotic stress, with low values representing reduced photosystem II function) highlighted that both *OsEPF1oe* lines maintained *F*_v_/*F*_m_ levels for at least a day longer than ‘IR64’ controls under drought conditions at 30°C, and the *OsEPF1oeS* line also at 40°C (Fig. 6b,f). When the plants were rewatered, 100% of *OsEPF1oe* plants grown at 30°C survived the drought period compared with only 50% of ‘IR64’ control plants (Fig. 6c,d). At 40°C, 50% of *OsEPF1oeS* plants survived the drought treatment, whereas all other plants died (Fig. 6g,h). Thus, reducing stomatal density leads to increased survival under severe drought, although at 40°C this was only apparent in *OsEPF1oeS* plants.

## Discussion

It is probable that 50% of rice crops already experience drought‐associated yield losses (Matsuda *et al*., 2016). Confronted with human population increases, climate change and water scarcity, there is an urgent need to reduce crop water use whilst maintaining photosynthesis, yield and heat tolerance at higher atmospheric CO_2_ concentrations (Ainsworth, 2008; Gago *et al*., 2014; Jagadish *et al*., 2015). To simulate future conditions, we conducted experiments at an elevated 450–480 ppm CO_2_ concentration. As reported previously in barley (Hughes *et al*., 2017), overexpression of *OsEPF1* in rice led to arrested stomatal development, resulting in reductions in stomatal density, stomatal index and, in some cases, stomatal size. Both in rice and barley, these phenotypic changes at the leaf surface led to increased drought tolerance by restricting water loss, both when water was plentiful and under drought conditions. As rice is typically grown in warm, bright tropical climates, we have further explored how plants with fewer stomata respond to high temperature (including under drought conditions) and at high light intensity to determine whether crops with reduced stomatal density could perform well in warmer, drier climates.

Infrared gas exchange analysis performed on plants with fewer than half the normal density of stomata showed no reductions in *A* at light intensities below 1000 μmol m^−2^ s^−1^ PAR. Despite increased plant temperatures and decreases in *A* under some growth conditions (e.g. when well watered at 30°C and 2000 μmol m^−2^ s^−1^ PAR), plants with reduced stomatal density consistently produced grain yields equivalent to, or greater than, ‘IR64’ controls when grown in growth chambers set to 1000 μmol m^−2^ s^−1^ light intensity. Furthermore, *OsEPF1oe* plants showed lower levels of water use at 30°C, only requiring *c*. 60% of the water used by controls when consumption was measured between weeks 4 and 5. Owing to their enhanced water conservation, *OsEPF1oe* plants could maintain transpiration for longer under drought, leading to an extended period of *A* and cooling relative to controls. Following drought during flowering (treatment 3), the *OsEPF1oeW* plants produced increased yield relative to both control and *OsEPF1oeS* plants. This suggests that a moderate reduction in stomatal density (*OsEPF1oeW*) rather than a severe reduction (*OsEPF1oeS*) was more beneficial under these conditions, perhaps because the recovery of large flowering plants after drought was hindered in the plants with the fewest stomata.

Reduced levels of transpiration and associated cooling, as seen in the well‐watered *OsEPF1oe* plants, might be expected to increase plant susceptibility to heat stress, but this is not what we observed. Our experiments growing plants at high temperature and elevated CO_2_ during the vegetative stage gave important insights into how crops with different stomatal density might perform in the future. We discovered that rice plants naturally increase the number of stomata that develop on leaves when grown at higher temperatures. Whilst *OsEPF1oe* lines did not do this, they were able to adapt effectively by increasing stomatal pore area. When assayed at high temperature and high light conditions this response enabled *OsEPF1oe* plants to increase *g*_s_ (and *A*) up to a level equivalent to ‘IR64’ controls.

Somewhat counterintuitively, when combined high growth temperature (40°C) and severe drought stress treatments were applied, half of the *OsEPF1oeS* plants were able to survive the harsh conditions when all other control and *OsEPF1oeW* plants died. We propose that the reduced stomatal density of *OsEPF1oeS* permitted improved water conservation before and during the drought, leading to an extended period of *g*_s_ and enhanced plant survival. Taken together with the results discussed earlier from drought experiments with more mature plants during flowering, this indicates that the optimum stomatal density required to perform well during and after episodes of drought is not always the same. Clearly, the optimization of stomatal characteristics to particular drought and temperature scenarios will require further investigation.

All our findings support the idea that cultivated rice may currently have higher *g*_s_ capacity than is required to maintain yields (Hu *et al*., 2006). In a future, warmer high‐CO_2_ world where water availability will decrease, altering stomatal density, size and or pore aperture could provide a solution that maintains yields and conserves water. Our data provide promise for future water‐use‐efficient rice that is more drought and heat tolerant. However, the effect of reducing stomatal density (and altering stomatal size and pore aperture) on field‐grown rice, experiencing other environmental fluctuations, remains untested. Based on our results, we suggest that reducing stomatal density may conserve water and protect, and in some cases even improve, rice yields under future climate conditions. Finally, by combining stomata‐related water use efficiency and drought tolerance with other stress‐responsive traits, we foresee further advances that could lead to the development of rice increasingly fine‐tuned for future warmer, drier, high‐CO_2_ climates.

## Author contributions

R.S.C., E.H.M., W.P.Q. and J.E.G. designed the study. R.S.C., E.L.H., J.S., T.F. and C.C.C. undertook the experiments with contributions from U.M. R.A.C., E.H.M., W.P.Q. and J.E.G. contributed materials and advice. R.S.C., X.Y., A.K.B., J.D. and A.B. constructed vectors for rice and Arabidopsis transformations, X.Y. and J.D. carried out rice transformations. R.S.C. and C.C.C. carried out Arabidopsis transformations. X.Y. and J.D. carried out Southern blot hybridization of the overexpressing lines. R.S.C., J.S., C.C.C., W.P.Q. and J.E.G. wrote the paper with comments from X.Y., E.L.H., U.M., T.F., J.D., R.A.C., A.B., E.H.M. and R.S. All authors read, commented on and approved the final version of the manuscript.

## Supporting information

Please note: Wiley Blackwell are not responsible for the content or functionality of any Supporting Information supplied by the authors. Any queries (other than missing material) should be directed to the *New Phytologist* Central Office.

**Fig. S1** Peptide sequence alignment and functional studies of the rice *OsEPF1* (*OSIR64_00232g011350*) gene.
**Fig. S2** Confocal microscopy imaging of stomata and underlying sub‐stomatal cavity formation and vein development in leaf 5 of 21‐d‐old rice plants.
**Fig. S3** Total guard cell area of IR64 control and *OsEPF1oe* plants grown at 30 and 40°C.
**Fig. S4** Leaf 5 analysis of gas exchange and photochemistry in *OsEPF1oe* plants.
**Fig. S5** Number of insertions and expression profiling in *OsEPF1* overexpressing lines.
**Fig. S6** *OsEPF1oe* plants droughted from 4 wk after germination.
**Fig. S7** *OsEPF1oe* biomass and grain yield.
**Fig. S8** Temperature and growth properties of IR64 control and *OsEPF1oe* plants grown at 40°C.
**Table S1** Values used for the calculation of anatomical *gs*_*max*_.
