# Supplementary material for: Rice with reduced stomatal density conserves water and has improved drought tolerance under future climate conditions
Source: New Phytol. 2018 Jul 24;221(1):371–84. doi: 10.1111/nph.15344 (PMC6492113; doi:10.1111/nph.15344)
Supplement: Supplementary file 1 — Fig. S1 Peptide sequence alignment and functional studies of the rice OsEPF1 (OSIR64_00232g011350) gene. Fig. S2 Confocal microscopy imaging of stomata and underlying sub‐stomatal cavity formation and vein development in leaf 5 of 21‐d‐old rice plants. Fig. S3 Total guard cell area of IR64 control and OsEPF1oe plants grown at 30 and 40°C. Fig. S4 Leaf 5 analysis of gas exchange and photochemistry in OsEPF1oe plants. Fig. S5 Number of insertions and expression profiling in OsEPF1 overexpressing lines. Fig. S6 OsEPF1oe plants droughted from 4 wk after germination. Fig. S7 OsEPF1oe biomass and grain yield. Fig. S8 Temperature and growth properties of IR64 control and OsEPF1oe plants grown at 40°C. Table S1 Values used for the calculation of anatomical gs max. [file NPH-221-371-s001.pdf]

New Phytologist

New Phytologist **Supporting Information**

Article title: **Rice with reduced stomatal density conserves water and has improved drought tolerance under future climate conditions**

Authors: Robert S. Caine, Xiaojia Yin, Jennifer Sloan, Emily L. Harrison, Umar Mohammed, Timothy Fulton, Akshaya K. Biswal, Jacqueline Dionora, Caspar C. Chater, Robert A. Coe, Anindya Bandyopadhyay, Erik H. Murchie, Ranjan Swarup, W. Paul Quick, Julie E. Gray

Article acceptance date: 10 June 2018

The following Supporting Information is available for this article:

Table S1 Values used for the calculation of anatomical gs_{max}.

Fig. S1 Peptide sequence alignment and functional studies of the rice *OsEPF1* (*OSIR64_00232g011350*) gene.

Fig. S2 Confocal microscopy imaging of stomata and underlying sub-stomatal cavity formation and vein development in leaf 5 of 21 day old rice plants.

Fig. S3 Total guard cell area of IR64 control and *OsEPF1oe* plants grown at 30°C and 40°C.

Fig. S4 Leaf 5 analysis of gas exchange and photochemistry in *OsEPF1oe* plants.

Fig. S5 Number of insertions and expression profiling in *OsEPF1* over-expressing lines.

Fig. S6 *OsEPF1oe* plants droughted from 4 weeks after germination.

Fig. S7 *OsEPF1oe* biomass and grain yield.

Fig. S8 Temperature and growth properties of IR64 control and *OsEPF1oe* plants grown at 40°C.

Table S1 Values used for the calculation of anatomical gs_{max}. Values measured from nail varnish peels of abaxial epidermal dental resin impressions of 21 day old rice leaves.

Temp. (°C)	Plant line	Pore depth (l, µm)	Aperture width (µm)	Potential aperture (a_{max}, µm^2)	Molecular volume of air (v)	Diffusivity of water (d)	Stomatal density (D, mm^{-2})
30	IR64 control	2.13±0.06	0.64±0.04	73.08±3.27	0.0249	0.0000259	165.10±5.11
	OsEPF1oeW	2.05±0.07	1.04±0.13	68.28±3.61			100.67±5.69
	OEPF1oeS	1.90±0.06	1.19±0.13	61.75±2.00			47.32±11.73
40	IR64 control	2.35±0.07	1.26±0.06	80.84±3.60	0.0260	0.0000324	226.85±21.27
	OsEPF1oeW	2.26±0.13	1.56±0.16	79.19±3.04			104.36±16.71
	OEPF1oeS	1.96±0.06	3.21±0.12	88.34±5.81			67.45±12.37

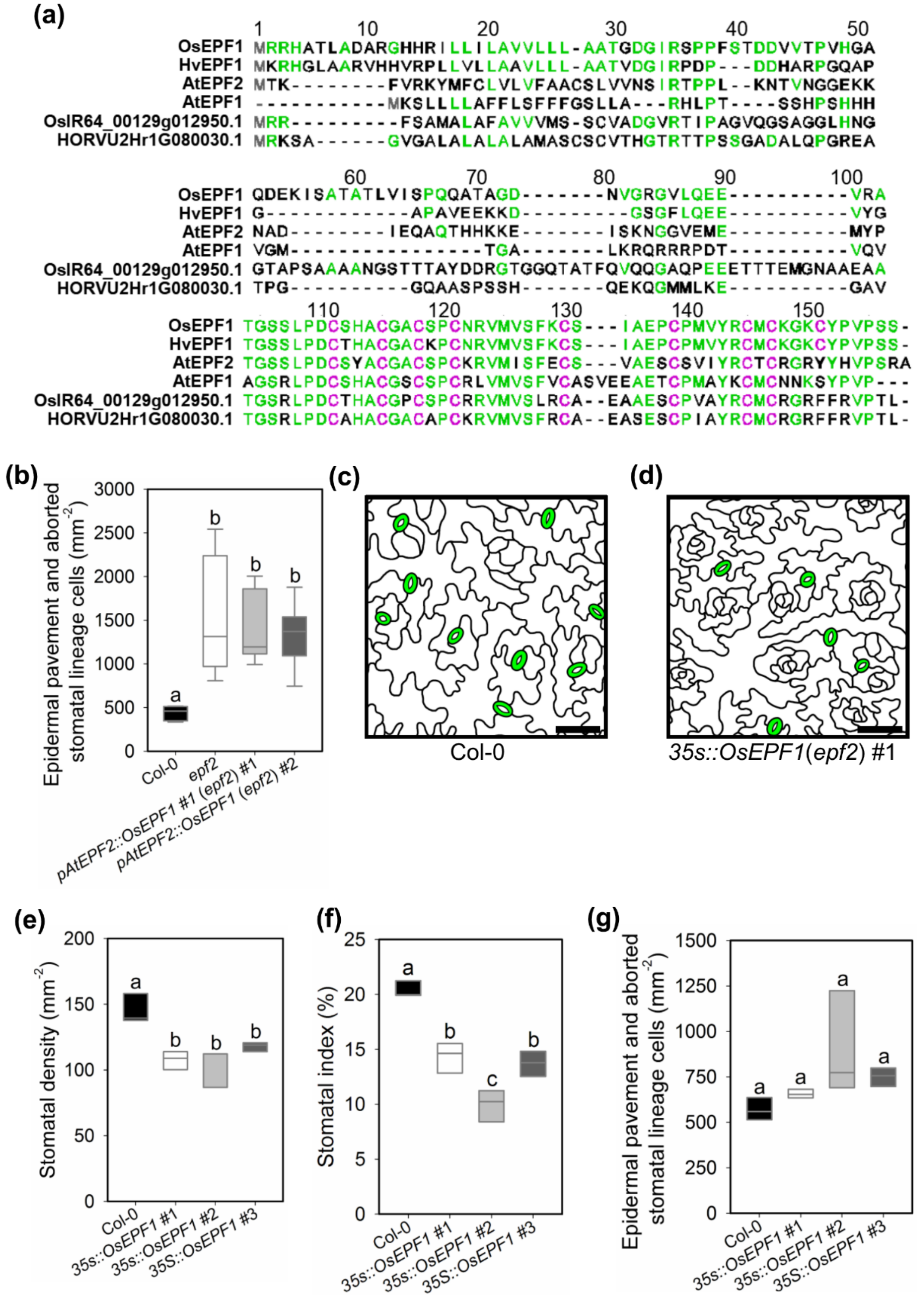

Fig. S1 Peptide sequence alignment and functional studies of the rice *OsEPF1* (*OSIR64_00232g011350*) gene. (a) Full peptide sequence alignment of closely related *EPF* genes in rice (*Oryza sativa* L. ssp. *Indica*), barley (*Hordeum vulgare*) and *Arabidopsis thaliana*.

Conserved cysteine residues (purple) and other shared amino acids (green). HvEPF1, HORVU2Hr1G116010.3; AtEPF2, AT1G34245.1; AtEPF1, AT2G20875.1. (b) Epidermal pavement and aborted stomatal lineage cells per mm^2 (non-stomatal cells) in Arabidopsis Col-0, *epf2* and two independent lines expressing the rice *OsEPF1* coding sequence under the native Arabidopsis *EPF2* promoter in the *epf2* background. The rice *OsEPF1* lines display a similar numbers of non-stomatal cells as *epf2* plants. (c and d) Tracing of images of 9 week old Arabidopsis leaf epidermis from (c) Col-0 control and (d) Col-0 line over-expressing *OsEPF1* cDNA under control of the CaMV*35S* promoter (Scale bars = 50µm). (e) Stomatal density, (f) index and (g) non-stomatal cells in three independent T3 *OsEPF1* over-expressing lines ($P < 0.05$). Stomatal density e and stomatal index f are both significantly reduced in *OsEPF1* over-expressing plants. The number of non-stomatal cells in g is showing a trend towards an increase although this is not significant. For graphs b,e,f, and g, horizontal lines within boxes indicate the median, and boxes indicate the upper (75%) and lower (25%) quartiles. Whiskers indicate the ranges of the minimum and maximum values. Lines marked with different letters in b, e and f are significantly different to Col-0 ($P < 0.05$, one-way analysis of variance). Due to unequal variance in b a Kruskal-Wallis One Way Analysis of Variance on Ranks was performed. For b, n=7 plants for e-g, n=3 plants.

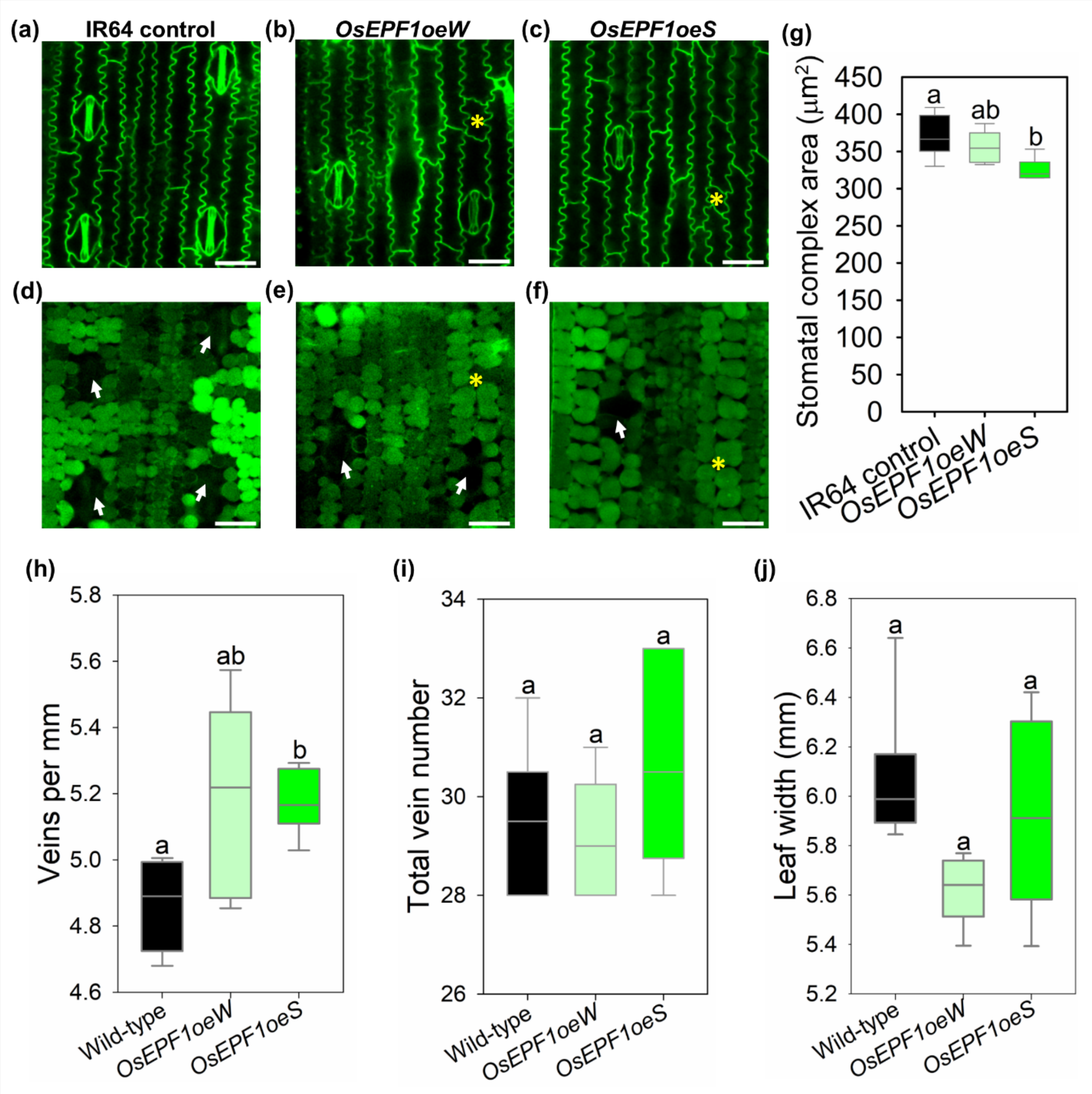

Fig S2 Confocal microscopy imaging of stomata and underlying sub-stomatal cavity formation and vein development in leaf 5 of 21 day old rice plants. Epidermal images of (a) IR64 control, (b) *OsEPF1oeW* and (c) *OsEPF1oeS* and (d, e and f) corresponding underlying tissue, with sub-stomatal cavities marked with white arrows. Aborted guard mother cells and corresponding underlying areas are marked with yellow asterisks. (g) Stomatal complex area. (h) Number of veins per millimetre in IR64 control, *OsEPF1oeW* and *OsEPF1oeS* lines. (i) Total vein number in leaves of the corresponding lines. (j) Maximum width of leaf 5 taken from 21 day old plants used for the analyses in h and i. For g-j, horizontal lines within boxes indicate the median, and boxes indicate

the upper (75%) and lower (25%) quartiles. Whiskers indicate the ranges of the minimum and maximum values. Lines marked with different letters are significantly different ($P < 0.05$, one-way analysis of variance). n=6 plants. Scale bars = 25μm.

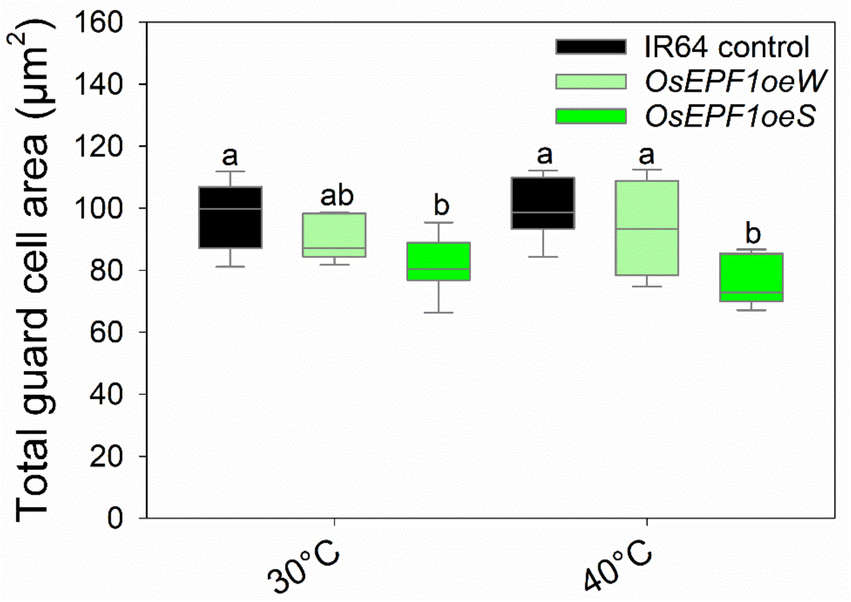

Fig S3 Total guard cell area of IR64 control and *OsEPF1oe* plants grown at 30°C and 40°C. Estimated from measurements on nail varnish peels of epidermal dental resin impressions. Horizontal lines within boxes indicate the median, and boxes indicate the upper (75%) and lower (25%) quartiles. Whiskers indicate the ranges of the minimum and maximum values. For 30°C n=7, for 40°C n=6 with 20 guard cell area measurements performed per plant. A two-way analysis of variance was carried out to detect significant differences between lines within and between treatments. Letters indicate significantly different mean values (P < 0.05).

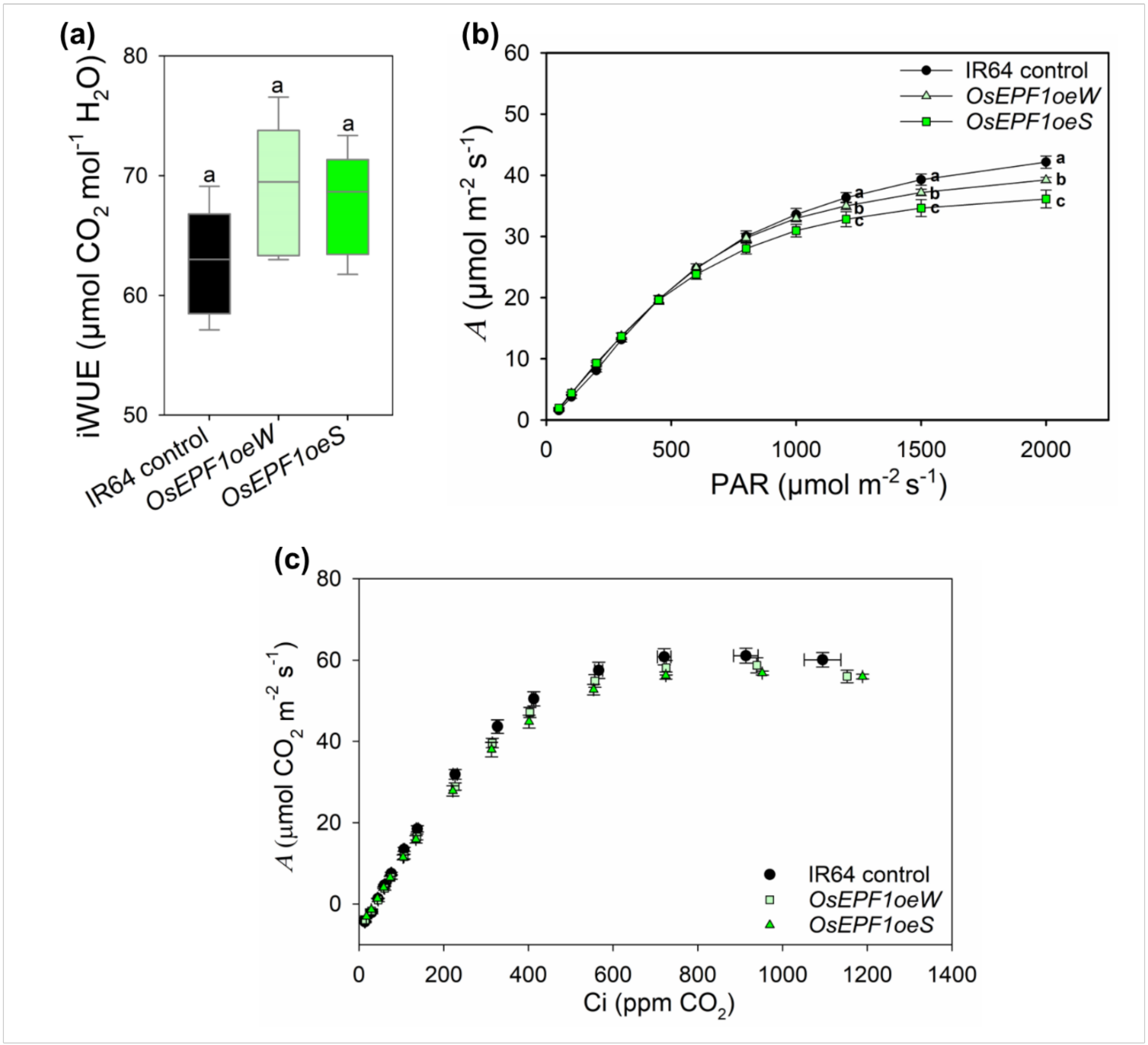

Fig S4 Leaf 5 analysis of gas exchange and photochemistry in *OsEPF1oe* plants. (a) Intrinsic Water Use Efficiency (iWUE). (b) Light curve of photosynthetic assimilation rate (*A*) at increasing light intensities. (c) CO_2 response curve of A to internal CO_2 concentration (*Ci*). IR64 control and *OsEPF1* over-expressing lines grown at 1000 µmol m^{-2} s^{-1} PAR. For a, horizontal lines within boxes indicate the median, and boxes indicate the upper (75%) and lower (25%) quartiles. Whiskers indicate the ranges of the minimum and maximum values. Letters indicate significantly different mean values ($P < 0.05$, one-way analysis of variance). Error bars indicate standard error of the mean (s.e.m.). a: n=7. b,c: n=6.

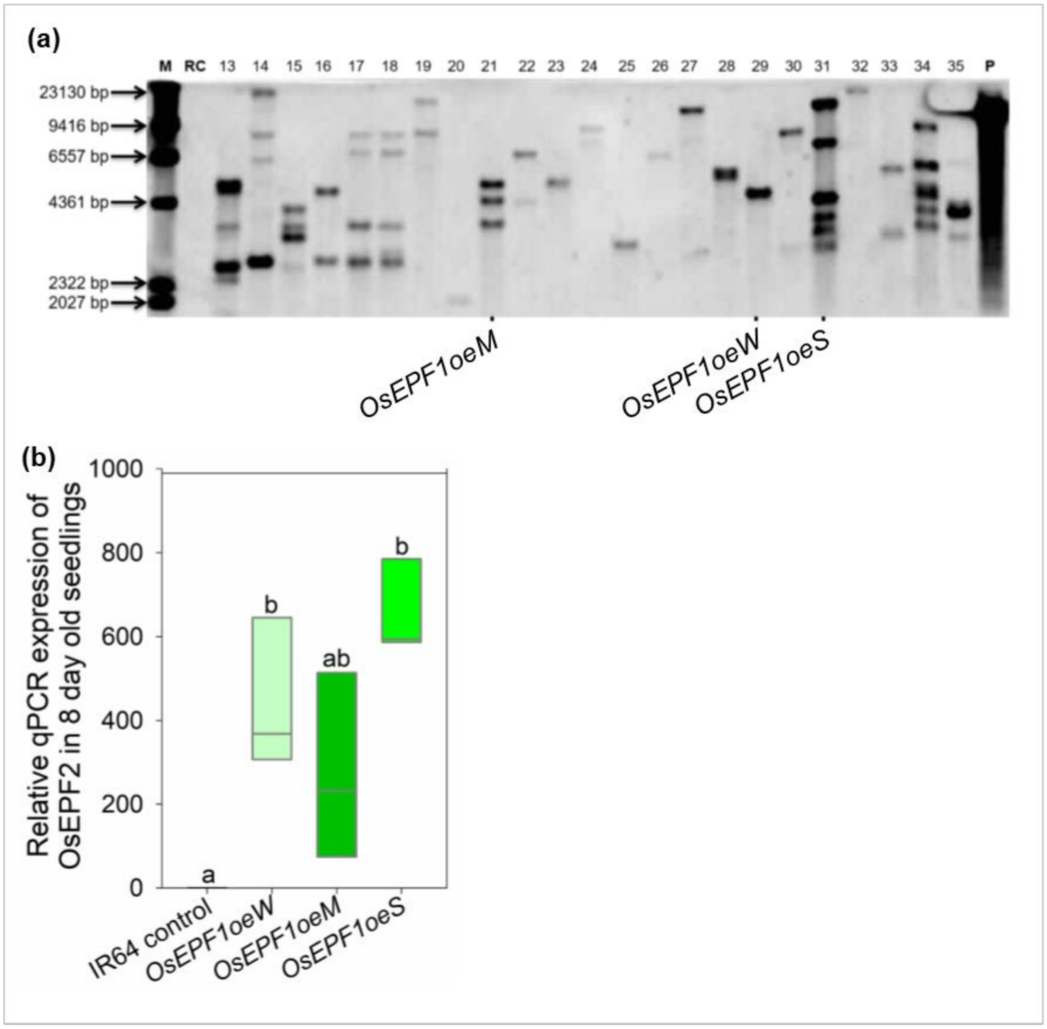

Fig. S5 Number of insertions and expression profiling in *OsEPF1* over-expressing lines. (a) Southern blot analysis of putative *OsEPF1* over-expressing lines showing one transgene insertion site in *OsEPF1oeW*, three in *OsEPF1oeM* and six in *OsEPF1oeS*. M denotes DNA weight marker, RC is an IR64 rice control without a resistance cassette, P is the positive plasmid control and numbers denote transgenic line number. (b) Relative qPCR analysis of 8 day old seedlings of *OsEPF1oeW*, *OsEPF1oeM* and *OsEPF1oeS* T2 plants reveals upregulation of *OsEPF1* in three independent lines. For b, horizontal lines within boxes indicate the median, and boxes indicate the upper (75%) and lower (25%) quartiles. Lines marked with different letters are significantly different. $P < 0.05$, one-way analysis of variance. For qPCR analysis n=3 biological replicates.

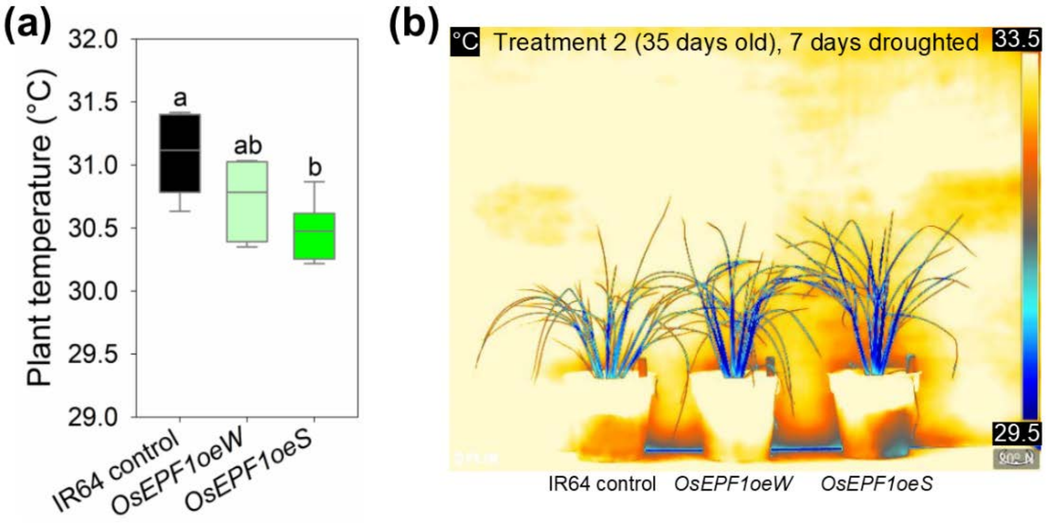

Fig S6 *OsEPF1oe* plants droughted from 4 weeks after germination. (a) Plant temperature, measured using an infra-red thermal imaging camera, of IR64 control, *OsEPF1oeW* and *OsEPF1oeS* lines on day 7 out of 9 days of drought. (b) Representative thermal image of plants used to collate data in (a). Dark blue represents the coolest areas. For a, horizontal lines within boxes indicate the median, and boxes indicate the upper (75%) and lower (25%) quartiles. Whiskers indicate the ranges of the minimum and maximum values. Lines marked with different letters are significantly different ($P < 0.05$, one-way analysis of variance).

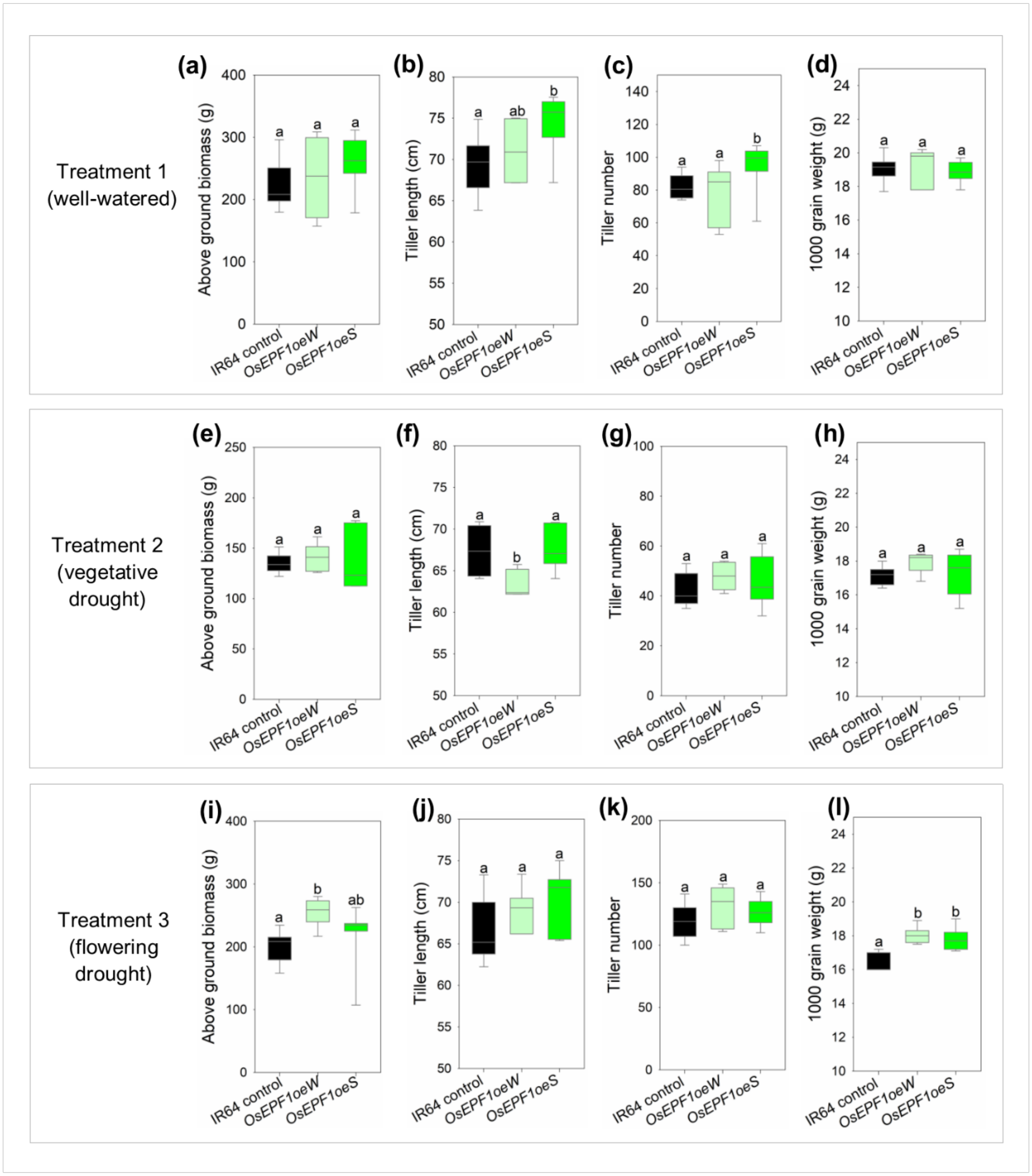

Fig S7 *OsEPF1oe* biomass and grain yield. (a-d) Treatment 1 – well watered. (e-h) Treatment 2 – vegetative drought. (i-l) Treatment 3 – flowering drought. (a,e,i) Above ground plant dry biomass. (b,f,j) Average length of five tillers of plants. (c,g,k) Number of tillers per plant. (d,h,l) 1000 grain weight. Horizontal lines within boxes indicate the median, and boxes indicate the upper (75%) and lower (25%) quartiles. Whiskers indicate the ranges of the minimum and maximum values. All

statistical tests performed using one-way analysis of variance with significant differences marked with different letters on individual graphs ($P < 0.05$).

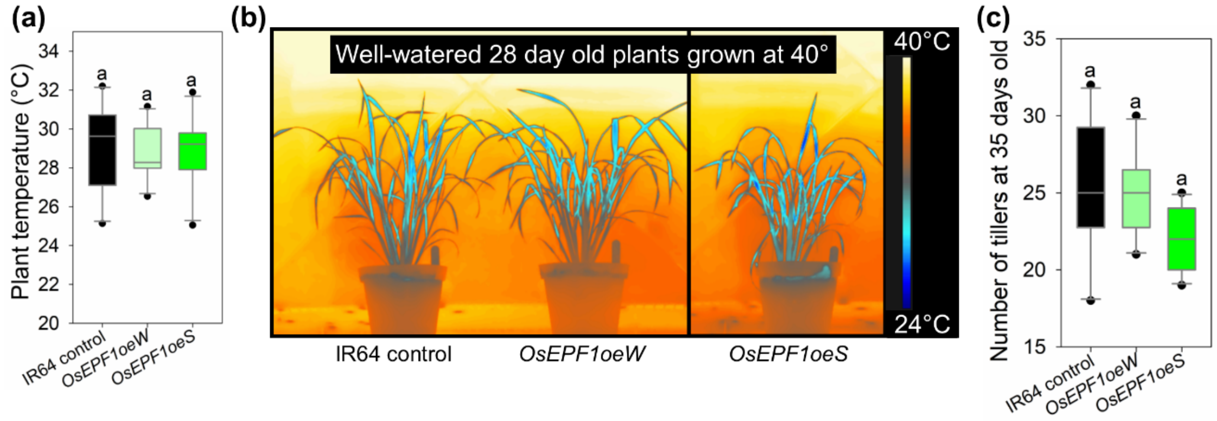

Fig S8 Temperature and growth properties of IR64 control and *OsEPF1oe* plants grown at 40°C. (a) Plant temperature, measured using an infra-red thermal imaging camera, of IR64 control, *OsEPF1oeW* and *OsEPF1oeS* lines of well-watered 28 day old plants. (b) Representative thermal images of plants used to collate data in a. Dark blue represents the coolest areas as per the scale bar. The image of *OsEPF1oeS* was taken from a separate image to that of the IR64 control and *OsEPF2oeW* plants as only two plants were used to catch individual images. Plants were imaged randomly with different combinations of genotype in each shot. (c) Number of tillers produced by 5 week old plants grown at 40°C. For a and c, Horizontal lines within boxes indicate the median, and boxes indicate the upper (75%) and lower (25%) quartiles. Whiskers indicate the ranges of the minimum and maximum values. Absolute outliers are marked by dots. No significant differences were detected when one-way analysis of variance testes were performed. Although not significantly different in tiller number, *OsEPF1oeS* plants did appear smaller when tiller number was counted. n=10.
